# Roles for epigenetics in wood formation and stress response intrees–from basic biology to forest management

**DOI:** 10.3389/freae.2025.1476499

**Published:** 2025-02-27

**Authors:** Andrew Groover

**Affiliations:** US Forest Service, Northern Research Station, Burlington, VT, United States

**Keywords:** abiotic stress, drought, epigenetics, forest management, wood formation

## Abstract

Annual model and crop species have been the subject of most epigenetic studies for plants. In contrast to annuals, forest trees persist on natural landscapes and experience environmental variation within and across seasons, years, and decades or even centuries. Most forest trees species are undomesticated and typically grown on variable landscapes with no irrigation or application of agricultural chemicals. Forest trees must thus rely on their inherent ability to alter growth and physiology to mitigate the effects of changing abiotic and biotic stressors. Like other plants, trees have mechanisms encoded in their genomic DNA sequence that can respond directly to stress events such as drought or heat. Hypothetically, it would be highly advantageous to join these mechanisms with a dynamic “memory” of past exposure to stress. It is now well established that annual model and crop plants can establish epigenetic-based memory of stress events that support more rapid and robust response to stress in the future. Here, evidence is discussed for epigenetic regulation and “memory” in two fundamental biological processes in trees, wood formation and abiotic stress response. Wood formation is an ideal trait for epigenetic research in trees, as wood formation is highly responsive to environmental conditions and includes multiple rapid developmental changes as cells adopt distinct fates within complex tissues. This is followed by a discussion of research needs that would provide the foundation for new epigenetic applications for forestry.

## Introduction

A key challenge in forest genetics is to explain the biological basis of adaptive trait variation that influences the ability of tree species or genotypes to persist and reproduce in different environments and in response to environmental variation over time. Variation in the DNA sequence among species and populations within species has generally been assumed to be responsible for the observed phenotypic differences in developmental, physiological and adaptive traits. But as discussed below, there is increasing evidence that DNA sequence is insufficient to explain all of the phenotypic variation for adaptive traits in forest trees. Epigenetic variation is likely a key component of the complex regulation of developmental and adaptive traits in trees not explained by DNA sequence variation and is the focus of this minreview.

Tree species are primarily outcrossing, characterized by highly heterozygous individuals and populations with high within-population DNA sequence variation (Porth and El-Kassaby, 2014). Obvious phenotypic variation can be observed across populations for adaptive traits, such as phenology traits measured in common garden experiments (e.g., Zohner and Renner, 2014). Explaining variation for developmental and adaptive traits within and among tree populations using genomic DNA sequence variation has been a primary goal of forest tree genomics but has seen modest success (Grattapaglia, 2022). The advent of new sequencing technologies has allowed for comprehensive assay of DNA sequence variation yet a significant percentage of observed phenotypic variation remains unexplained in many tree genomic studies (Grattapaglia, 2022), a so called “missing” heritability problem (Zuk et al., 2012). A number of technical issues including inability to include higher order epistatic interactions, structural variation or rare alleles into models may explain some of the shortcoming (Matthews and Turkheimer, 2022), but it also raises the question if additional biological mechanisms other than DNA sequence are contributing to phenotypic variation?

Classically, epigenetic variation referred to heritable phenotypic variation that is not determined by genomic DNA sequence variation (Berger et al., 2009). Since the identification of molecular mechanisms underlying epigenetic variation, however, this definition has broadened to also include epigenetic phenomenon that influence development or response to the environment within individuals regardless of heritability (Webster and Phillips, 2024). At a molecular level, epigenetic chemical modifications of the genome include DNA methylation, various modifications of histones, and the action of non-coding RNAs (Martienssen et al., 2008). DNA methylation plays multiple roles in plants including regulation of transposable elements and modifying expression of genes (Martienssen and Colot, 2001). Acetylation, methylation, ubiquitination and phosphorylation of specific histone amino acid residues can change chromatin conformations to promote or repress gene expression in plants (Zhao et al., 2019). For example, trimethylation of lysine 4 of histone H3 (H3K4me3) promotes gene expression, while trimethylation of lysine 27 of histone H3 (H3K27me3) represses gene expression (Niu et al., 2018). At the RNA level, small RNAs including microRNAs (miRNAs), heterochromatic small interfering RNAs, and secondary small interfering RNAs can affect developmental and stress responses through regulation of post transcriptional gene silencing (PTGS), DNA methylation, and transposon activity (Zhan and Meyers, 2023).

An increasing foundation of research points to roles for epigenetic regulation of developmental and adaptive traits in model angiosperm annual plants (Chang et al., 2020; Hannan-Parker et al., 2022). For example, Arabidopsis grown for five generations under imposed selection showed heritable changes in phenotypes that were not attributable to changes in DNA sequence but were associated with significant changes in DNA methylation (Schmid et al., 2018). A survey of methylomes of over 1,000 Arabidopsis natural accessions found that methylation contributes to adaptive trait variation in natural populations, with methylation variation associated with environmental response genes (Kawakatsu et al., 2016). Specific drought-responsive genes in Arabidopsis have increased H3K4me3 and H3K9Ac levels associated with their increased expression during drought (Kim et al., 2012). Abscisic acid (ABA) is central to plant drought response, and H3K4me3 modification of the *NINE CIS-EPOXYCAROTENOID DIOXYGENASE 3* (*NCED3*) gene encoding a key enzyme in ABA biosynthesis is associated with increased expression during drought response (Ding et al., 2011).

Hypothetically, epigenetic regulation has the potential for significant impacts on forest tree adaptive traits beyond what is seen in annuals. For example, the ability to establish a memory of past stress could allow individual long-lived trees to mount more robust responses to future stress within or across growing seasons, or even across generations. Epigenetic mechanisms provide the molecular basis of such memory as well as for plasticity within individual trees (Miryeganeh and Armitage, 2024; Lloyd and Lister, 2022). Across generations, demonstrating transgenerational epigenetic inheritance is inherently difficult (Heard and Martienssen, 2014) and convincing examples in trees with long generation times are thus limited. In one example, grafting experiments in Norway spruce demonstrated that the same genotype could produce seedlings with varying phenology traits (Yakovlev et al., 2012), which reflected differences in day length and temperature experienced by the maternal environment during post-meiotic megagametogenesis and seed maturation (Skrøppa et al., 2007). Similarly, maternal environment was found to influence resistance to a *Fusarium* pathogen in offspring (Vivas et al., 2013). The molecular basis of these presumed epigenetic traits is not known, however. Nonetheless, it is intriguing to imagine a single tree that persists on a landscape for decades or even hundreds of years might produce an array of epigenetically conditioned gametes across years in response to environmental change.

What follows is a discussion of some of the technical challenges of measuring epigenetic variation in trees, which is followed by examples of epigenetic regulation of wood formation and adaptive traits in trees. At the end of this review, important research questions around epigenetics in forest trees and how they affect forest management and conservation are presented.

## Measuring epigenetic variation in trees

Forest trees are experimentally challenging for epigenetic studies when compared to annual model or crop plants. Most forest trees have an extended juvenile period before becoming reproductively mature, limiting cross generational studies. Additionally most tree species are outcrossing and highly heterozygous, making it difficult to distinguish the relative effects of segregating DNA sequence variation *versus* epigenetic variation within pedigrees or populations. One approach for non-transgenerational studies has been to take advantage of poplar species that can be clonally propagated and for which genomic sequence is available. For example, clonally propagated poplar hybrid genotypes were exposed to contrasting environments and subsequently grown in a common garden experiment where they were assigned to either well-watered or drought treatments (Raj et al., 2011). Differences in transcript abundance and DNA methylation were noted in response to drought for genetically identical clones sourced from contrasting environments, suggesting an epigenetic memory of prior environmental conditions (Raj et al., 2011).

Trees are phylogenetically diverse and include both gymnosperm and angiosperm species separated by 300M years of evolution (Groover, 2005). There are striking differences between angiosperm and gymnosperm genomes that could affect epigenetic mechanisms. Conifer genomes are characterized by exceptional size, genes with large introns, unique evolutionary history of repeat sequences, and a lack of recent genome duplications events (Wan et al., 2022). Angiosperm tree genomes are highly diverse and reflect past whole genome duplication and hybridization events, have frequent structural variation, and have more compact gene structures (Benton et al., 2022). A limited number of forest trees have complete genome sequences supporting approaches like Chromatin Immunoprecipitation sequencing (ChIP-seq) capable of surveying chromatin marks, most notably poplar species (Liu et al., 2015). Differences between and within gymnosperm and angiosperm lineages for variation in epigenetic variation and responses is a largely unexplored area of research. Additionally, there is a dearth of information regarding the dynamics of epigenetic variation across difference timescales in trees, including how quickly new epigenetic states are established, how long they persist, and what environmental cues effectively reset or change epigenetic states. Similarly, it remains uncertain if different types of epigenetic marks might be more important for specific types of environmental variation, or if different marks might be associated with different timescales or more quickly erased marks. However, as of 2020, a total of 83 publications addressed some aspect of epigenetic regulation in trees, reflecting a young but growing body of research (Amaral et al., 2020).

Next is a discussion of two examples of epigenetic regulation of traits that are central to the understanding of forest tree biology and evolution, that also have relevance to the management and conservation of trees in response to environmental variation and climate change. These brief examples are not intended to be exhaustive reviews.

## Examples of epigenetic regulation of wood formation

Wood formation in trees is developmentally plastic and highly responsive to environmental conditions. For example, angiosperm trees make more numerous and small diameter vessels to resist embolism under heat and drought conditions (Rodriguez-Zaccaro and Groover, 2019). Additionally, wood formation is developmentally dynamic, with maintenance of dividing stem cells within the cambium balanced with the coordinated differentiation of daughter cells into the different cell types of wood. Multiple lines of evidence point to a significant role for epigenetic regulation of both developmental dynamics and integration of environmental cues during wood formation. For example, the expression of genes encoding WOX-like transcription factors that regulate cell division in the vascular cambium (Kucukoglu et al., 2017) is modulated by the zinc finger protein PtrVCS2 through hypoacetylation of H3K9, H3K14 and H3K27 marks in the *PtrWOX4* promoter (Dai et al., 2023). At a genomic scale, Chip-seq profiling of H3K4me3 and H3k27me3 marks of wood forming tissues in eucalyptus found a correlation between these marks and genes involved in lignification, and could be used to predict ∼50% of variation in transcript abundance (Hussey et al., 2017). In contrast, in genome wide surveys in poplar DNA methylation was found to have only marginal effects on the expression of genes and pathways associated with wood formation (Zhang et al., 2020). However, DNA methylation profiling data from a population of balsam poplar was used to predict significant percentages of phenotypic variation for wood density, soluble lignin and cell wall carbohydrate (Champigny et al., 2020). From these studies it might be speculated that, histone-related marks can have direct and dramatic effects on expression of individual genes and phenotypes, while DNA methylation may contribute to phenotypic variation that is only revealed through more global quantitative analyses.

Roles for non-coding RNAs in wood formation are emerging from studies cataloging small RNA expression using RNA sequencing. For example, more than 2,000 miRNAs were expressed in different stages of wood development in poplar (Wang et al., 2021). Regulatory roles for miRNAs in wood formation were illustrated by dramatic effects on wood patterning and cambium function in transgenic poplar expressing a miRNA-resistant form of the Class III HD ZIP transcription factor, popREVOLUTA (Robischon et al., 2011).

## Examples of epigenetic regulation of drought stress response in trees

The stress hormone ABA is central to drought response in plants, including in trees (Yu et al., 2019). Histone modifications have been shown to play a role in ABA-mediated gene expression and abiotic stress response phenotypes in annual plants (Shi et al., 2024). In poplar, specific NAC transcription factor encoding genes (*PtrNAC006*, *PtrNAC007*, and *PtrNAC120*) are induced by drought and have ABA-response element (ABRE) motifs in their promoters (Li et al., 2019). Genome wide profiling correlated H3K9ac marks in the promoters of these NACS with their expression, which is modified by the ABRE-binding protein PtfAREB1 that recruits histone acetyltransferase to the NAC promoters (Li et al., 2019). Further, downregulation of these NACs resulted in drought hypersensitive phenotypes (Li et al., 2019).

The role of DNA methylation during drought stress response was evaluated in poplar through RNA interference (RNAi) downregulation of *DECREASED IN DNA METHYLATION 1* (*DDM1*) which encodes a highly conserved chromatin remodeling ATPase required for DNA and histone methylation (Lee et al., 2023; Vongs et al., 1993). Downregulation of poplar *DDM1* resulted in trees that were modestly more resistant to drought induced reduction in height growth and to air embolism of xylem (Sow et al., 2021). The *DDM1* downregulated trees also showed changes in methylation patterns, gene expression, and hormone levels (Sow et al., 2021), although specific mechanisms connecting methylation changes to phenotypes remain uncertain.

## Research needs and applications for forestry

Epigenetics has the potential for multiple practical applications in forest management (Amaral et al., 2020). If epigenetic variation could be selected or manipulated in parallel with traditional breeding, this could ultimately increase the gains realized for target traits within a breeding cycle. Research is needed to determine the causal relationships, heritability and magnitude of effect of specific chromatin marks on phenotypic traits to fully evaluate the potential of integrating epigenetic approaches into tree breeding. Additionally, the most effective methods of manipulating epigenetic variation in trees must still be established. However, simply understanding epigenetic effects on breeding efforts could be useful even in the absence of understanding the underlying mechanisms. For example, previous research found significant differences in performance of Norway spruce seed grown from clonally propagated parent trees at different seed orchard locations, showing the potential for epigenetic effects on reforestation success (Skrøppa et al., 2007). This raises the question of whether collecting seed from orchards or wild trees in less stressful environments could result in seedlings that are epigenetically maladapted to more stressful outplanting environments during reforestation.

Nursery production of tree seedlings presents the opportunity to create useful epigenetic variation through stress priming. In general, outplanting is a critical stage of reforestation, as seedlings have small root systems and limited water/nutrient reserves to withstand heat and drought. Stress priming uses controlled exposure to stress or to chemical or hormonal surrogates and can result in more rapid and robust response to future stress (Hilker and Schmülling, 2019). While variations of stress priming have previously been applied to tree nursery seedlings (e.g., “drought hardening”), in general they have had limited effectiveness (Puértolas et al., 2024) and were not developed in conjunction with research capable of evaluating biological mechanisms. New genomics-based research could examine the mechanistic response to different chemical, hormonal or cultural stress priming treatments on relevant epigenetic and physiological mechanisms to optimize treatments. Effective stress priming could be applied to seedlings in nursery settings or even through seed priming to increase resilience to outplanting stressors.

Non-coding RNAs are attractive targets for new applications for forestry. For example, RNA-based methods could be applied to one of the major threats to forest, invasive pathogens and insects (e.g., Lovett et al., 2016). Spray Induced Gene Silencing (SIGS) uses exogenously applied double stranded RNAs (dsRNAs) designed to trigger RNAi-based silencing of essential genes in pathogen or insect pests. This approach develops non-GMO biopesticides that are highly specific to the target pest and are non-toxic to the environment. Recent examples of effective SIGS in trees include myrtle rust (Degnan et al., 2023), pine pith canker (Bocos-Asenjo et al., 2025), and emerald ash borer (Rodrigues et al., 2018). SIGS could also potentially be used to downregulate genes in trees, for example, to more rapidly evaluate candidate genes for resistance or susceptibility to invasive pests. Beneficial alleles of candidate genes that are determined effective could then be selected through molecular-guided breeding. Genes with negative effects on phenotypes could also be downregulated through GMO-based RNAi, or through GMO-free CRISPR based gene editing (e.g., Yang et al., 2023).

Epigenetics likely play roles in other fundamental aspects of tree biology that affect developmental and adaptive traits. Hybridization and polyploidy are important features of many angiosperm tree species, and are known to include epigenetically-mediated interactions between parental genomes and complex changes in gene expression in model annuals (reviewed in Duarte-Aké et al., 2023). Gene copy number variation and structural variation are frequent in natural poplar populations (Pinosio et al., 2016) and have been shown to affect various quantitative traits in experimental populations (Bastiaanse et al., 2019). Such variation is correlated with epigenetic marks affecting gene expression and adaptive divergence (Shi et al., 2024). Similarly, epigenetic regulation of transposable elements can affect both mobilization and expression of nearby genes (Weil and Martienssen, 2008).

Moving forward, experiments capable of quantifying the magnitude of effects of epigenetic variation on developmental and adaptive traits need to be conducted. Importantly, such studies must move beyond simply correlating epigenetic molecular marks with traits and employ experimental designs capable of partitioning DNA sequence variance and epigenetic variance, to measure the magnitude of effect of epigenetic variation on phenotypic traits. As previously mentioned, tree species that can be clonally propagated may prove useful for such studies. A largely unexplored but critical area for trees is to determine how different levels or length of exposure to stress result in establishment of new epigenetic states, and how long these states last or are reset for different traits. Experiments focused on the dynamics of epigenetic programming could be overlaid with surveys of different epigenetic marks to determine which marks are the most important for different traits or associated with different dynamics or timescales. Additionally, variation among tree species for basic epigenetic processes and dynamics should be explored. Whether there might be fundamental differences between angiosperm and gymnosperm trees in epigenetic processes is largely unknown. And whether there might be significant differences among trees with different lifestyles is unknown. For example, might epigenetic states be more dynamic in short-lived pioneer species *versus* longer lived species? Finally, epigenetics could be a major factor determining how trees respond to increasing variation in weather extremes associated with climate change. Understanding the epigenetic regulation of tree developmental and adaptive traits might thus provide useful new insights into expectations and management tools for mitigating effects of climate change on forests.
